# A qualitative investigation of the experiences of patients living with antiphospholipid antibodies

**DOI:** 10.1177/09612033241265545

**Published:** 2024-07-24

**Authors:** Francesca S Cardwell, Alexandra O Kobza, Susan J Elliott, Paul S Gibson, Nancy Soliman, Leslie Skeith, Ann E Clarke, Megan RW Barber

**Affiliations:** 1 Department of Geography & Environmental Management, 8430 University of Waterloo, Waterloo, ON, Canada; 2 Division of Rheumatology, Cumming School of Medicine, 2129 University of Calgary, Calgary, AB, Canada; 3 Department of Obstetrics & Gynecology, Cumming School of Medicine, 2129 University of Calgary, Calgary, AB, Canada; 4 Division of Hematology and Hematological Malignancies, Cumming School of Medicine, 2129 University of Calgary, Calgary, AB, Canada

**Keywords:** Systemic lupus erythematosus, antiphospholipid antibodies, antiphospholipid syndrome, qualitative

## Abstract

**Objective:**

Substantial morbidity and mortality affect those with antiphospholipid antibodies (aPLs) and antiphospholipid syndrome (APS), yet patient experiences remain poorly understood. This research investigated patient experiences of aPL/APS diagnosis; effects on daily life; and healthcare and treatment.

**Methods:**

Patients aged ≥18 years with APS per the Revised Sapporo criteria or with ≥1 positive aPL on ≥2 occasions were recruited from a Canadian multidisciplinary APS clinic to participate in semi-structured in-depth interviews. Interviews were conducted virtually and transcribed verbatim for subsequent thematic analysis.

**Results:**

Twenty-one patients with aPLs/APS participated; 95.2% were female, mean (SD) age was 45.6 (15.0) years. Most (71.4%) had APS, and 71.4% had aPLs/APS with SLE. Results are presented around patient experiences of aPL/APS diagnosis, effects on daily life, and healthcare and treatment. Participants described medical complications/physical symptoms and the healthcare, lifestyle, and emotional impacts experienced around the time of aPLs/APS diagnosis. In addition to the physical and psychosocial impacts of living with aPLs/APS, patients reported modified leisure activities, altered employment trajectories, and positive and negative impacts on relationships. Impacts on family planning were also a critical component of the aPL/APS lived experience; participants shared experiences of miscarriage, other pregnancy complications, and medication-related challenges (e.g., with low-molecular-weight heparin injections). Challenging aspects of aPL/APS healthcare and treatment were also discussed, particularly related to the lifestyle, physical, and emotional burden of medication use. Although a lack of resources was described, participants expressed trust in healthcare providers when making management decisions or when seeking information. Suggestions for resources included the need for additional medication-related information, examples to help contextualize management behaviours, and additional information for those with aPLs/APS without SLE.

**Conclusion:**

Patients highlighted how the diverse manifestations of aPLs/APS, accentuated by management-related challenges, impose considerable physical and psychosocial burdens. Results will inform the development of patient resources aligned with patient priorities.

## Introduction

Antiphospholipid syndrome (APS) is an autoimmune condition defined by thrombosis or pregnancy morbidity and the presence of persistently positive antiphospholipid antibodies (aPLs).^1,2^ APS can develop in patients with no underlying systemic autoimmune condition or in those with overarching autoimmune conditions, primarily systemic lupus erythematosus (SLE). Approximately 40% of SLE patients have aPLs^3^; of these, up to 50%–70% may develop APS after 20 years of follow-up.^3,4^ SLE patients with APS have a worse prognosis^5^ than those without APS.^3^

Individuals with aPLs are at risk of developing thrombotic events^4^ and pregnancy complications and loss.^6^ Patients with thrombotic APS can experience arterial, venous, or small vessel thrombosis presenting most commonly as stroke, myocardial infarction, pulmonary embolism, or deep vein thrombosis.^4^ After a first unprovoked event, the annual recurrence rate without anticoagulation is 19%-29%,^4,7^ so indefinite anticoagulation (e.g., with warfarin) is often recommended.^8,9^ Those with obstetrical complications can experience recurrent early or late pregnancy losses, placenta-mediated pregnancy complications including early/severe pre-eclampsia, fetal growth restriction, and preterm delivery.^6,10^ Even with standard of care treatment (low-molecular-weight heparin (LMWH) and/or acetylsalicylic acid (ASA)), a high rate of pregnancy loss and obstetrical complications remain.^10–14^ Despite limited evidence supporting LMWH/ASA,^15–20^ previous qualitative research demonstrated that women often prefer taking action by self-injecting LMWH throughout pregnancy even in the absence of a guaranteed good outcome.^21^

The diverse manifestations of aPLs/APS can considerably impact patients, but patient experiences of aPLs/APS remain poorly understood. Quantitative studies^22–26^ have reported quality of life impacts in APS patients; one study assessed the psychosocial burden of APS, but the sample size was small.^27^ While existing qualitative studies report experiences of fetal loss,^28,29^ how patients and physicians navigate the LMWH and/or ASA decision-making process in pregnancy,^21^ patient perceptions of APS care,^30^ and fatigue,^31^ the broad lifecosts (i.e., totality of social, economic, physical, emotional and wellbeing impacts^32^) of living with aPLs/APS have not been documented. This study used a social constructionist approach to investigate patient lived experiences of aPL/APS (1) diagnosis; (2) effects on daily life; and (3) healthcare and treatment.

## Methods

Patients aged ≥18 years with APS per the Revised Sapporo criteria for APS^1^ or ≥1 positive aPL (medium to high positive anticardiolipin and/or medium to high positive anti-beta2-glycoprotein 1 and/or positive lupus anticoagulant) on ≥2 occasions were purposefully recruited from a Canadian multidisciplinary SLE/APS clinic (care provided by a rheumatologist, immunologist, and hematologist) to participate in semi-structured in-depth interviews. Participants were identified by the clinical team and approached in clinic or by telephone; we purposefully sampled patients with varied demographic backgrounds (e.g., age, race/ethnicity) and disease characteristics (e.g., with/without SLE per 1997 American College of Rheumatology^33^ or 2012 Systemic Lupus International Collaborating Clinics^34^ criteria) to ensure maximum variation of experiences. Recruitment continued until thematic saturation was reached.

Prior to recruitment, this study received ethics approvals from a Research Ethics Committee at the University of Waterloo, and the Conjoint Health Research Ethics Board at the University of Calgary. Semi-structured in-depth interviews were conducted virtually from March-July 2023 and lasted an average of 45 min. All interviews were conducted by the first author (female), an experienced qualitative researcher with 15+ years of experience. Participants were told the interviewer’s name, affiliation, and purpose of the research prior to the interview. Interviews were audio recorded with consent, and transcribed verbatim for subsequent thematic analysis using NVivo software independently by the first and second authors.

The interview guide (available upon request) was informed by our previous research on patient experiences of SLE, lupus nephritis (LN), and pregnancy and APS,^21,32,35^ and was designed to explore experiences of diagnosis, daily life, healthcare and treatment, and aPLs/APS resources/supports. Themes and sub-themes emerged from the data deductively (i.e., based on research objectives) and inductively (i.e., derived from the data). The first author developed an initial theme code set which was reviewed by the second and third authors. During proofing, the theme code set was refined by the first author and a final theme code set was used to code all transcripts using line-by-line open coding. The first and second authors discussed findings and discrepancies in coding to ensure consistency in the analysis. No participants dropped out of the study following the interviews.

A trained patient partner was involved in the research protocol design, and they reviewed the interview guide prior to the start of interviews.

## Results

### Participant summary

Twenty-one patients with aPLs/APS were interviewed; 95.2% were female, 19.1% were of non-white race/ethnicity, mean (SD) age was 45.6 (15.0) years, and mean (SD) aPL/APS disease duration was 6.9 (6.3) years (Table 1). Most (71.4%) participants had APS, 28.6% had aPLs; 71.4% had aPLs/APS with SLE. Participants were on hydroxychloroquine (71.4%), anticoagulants (52.4%), antiplatelets (61.9%), and immunosuppressants (33.3%) in the year prior to interview completion. Most (90.5%) participants had other co-morbidities, including cardiovascular disease (33.3%), hypertension (33.3%), malignancy (23.8%), thyroid disease (23.8%), and depression and/or anxiety (19.1%) (Table 1).

**Table 1.** Patient characteristics.

Characteristic	Total sample
All aPLs/APS, *n* (%)	21 (100%)
APS per sapporo criteria^a^, *n* (%)	15 (71.4%)
APS with SLE, *n* (%)	9 (42.9%)
Thrombotic, *n* (%)	9 (42.9%)
Primary APS, *n* (%)	6 (28.6%)
Thrombotic, *n* (%)	1 (4.8%)
Obstetric, *n* (%)	2 (9.5%)
Thrombotic & obstetric, *n* (%)	3 (14.3%)
≥1 positive aPL on ≥2 occasions, *n* (%)	6 (28.6%)
aPLs with SLE, *n* (%)	6 (28.6%)
Age (years) at time of interview, mean (SD)	45.6 (15.0)
aPL/APS disease duration^b^ (years) at time of interview, mean (SD)	6.9 (6.3)
SLE disease duration (years) at time of interview, mean (SD) (n = 15)	15.0 (13.8)
Female, *n* (%)	20 (95.2%)
Self-reported non-white race/ethnicity, *n* (%)	4 (19.1%)
Patient co-morbidities	
Cardiovascular disease	7 (33.3%)
Hypertension	7 (33.3%)
Malignancy	5 (23.8%)
Thyroid disease	5 (23.8%)
Depression/anxiety	4 (19.1%)
Cerebrovascular disease	3 (14.3%)
Chronic pain	3 (14.3%)
Renal disease	3 (14.3%)
Osteoarthritis	2 (9.5%)
Diabetes	1 (4.8%)
Osteoporosis	1 (4.8%)
APS with SLE: medications taken in the past year^c^, % (*n* = 9)	
Hydroxychloroquine, *n* (%)	9 (100%)
Anticoagulants^d^, *n* (%)	8 (88.9%)
Antiplatelets, *n* (%)	3 (33.3%)
Immunosuppressants^e^, *n* (%)	3 (33.3%)
Primary APS: medications taken in the past year^c^, % (*n* = 6)	
Hydroxychloroquine, *n* (%)	2 (33.3%)
Anticoagulants^f^, *n* (%)	3 (50.0%)
Antiplatelets, *n* (%)	5 (83.3%)
Immunosuppressants^e^, *n* (%)	0 (0.0%)
aPLs with SLE: medications taken in the past year^c^, % (*n* = 6)	
Hydroxychloroquine, *n* (%)	4 (66.7%)
Anticoagulants, *n* (%)	0 (0.0%)
Antiplatelets, *n* (%)	5 (83.3%)
Immunosuppressants^e^, *n* (%)	4 (66.7%)

^a^Includes three patients that do not strictly fulfil Sapporo criteria, as the interval between thrombotic/obstetric event and positive aPL measurement was greater than 5 years.

^b^Calculated using date of APS diagnosis for those with APS, and with date of first positive aPL for those with aPLs.

^c^Categories are not mutually exclusive.

^d^One participant had two provoked venous thrombotic events and was not placed on long term anticoagulation with warfarin.

^e^Immunosuppressants include: azathioprine, belimumab, methotrexate, mycophenolate mofetil or mycophenolic acid, and TNF-inhibitors.

^f^Two participants were not anticoagulated because they had previous obstetric events. One participant was not anticoagulated because although they had a myocardial infarction the etiology was uncertain; it may have been due to either coronary artery dissection or coronary artery disease.

### Diagnosis

**Figure 1.**
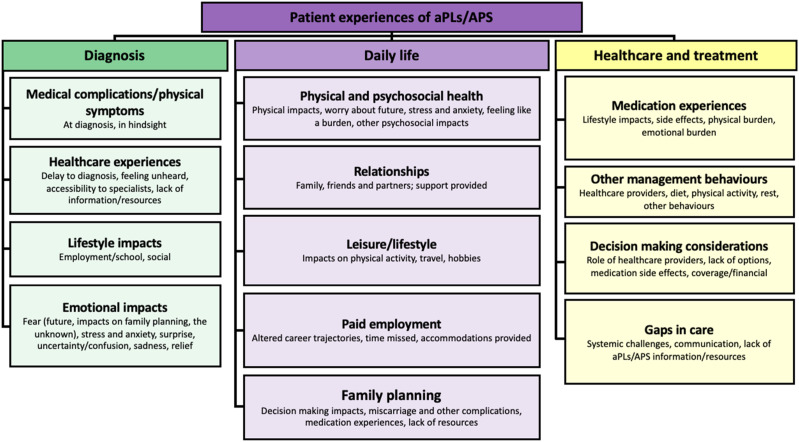
Overview of themes emerging through qualitative analysis.

Interviews began with a discussion of aPL/APS diagnosis. The primary themes were: medical complications/physical symptoms, healthcare experiences, lifestyle impacts, and emotional impacts (Figure 1; Table 2; Supplemental Table 1).

**Table 2.** Experiences of diagnosis.

Primary themes	Sub-themes	Illustrative quotes
Medical complications/physical symptoms	At diagnosis	I found out with a bang. I had a submassive pulmonary embolism, complicated by two cardiac arrests (#1; APS + SLE)
		I had some really bad chest pain, indicative of maybe a heart attack, and went into Emergency. And they said, you’re lucky you came when you did… you have a double embolism, one in each lung. So that’s kind of what started it. (#7; APS)
		I was having minor headaches for over a year… I eventually get my CT, and they’re like, oh, so there’s a blood clot in your jugular vein… then after that they ran some blood tests. (#19; APS + SLE)
	In hindsight	It just gave me better knowledge about why I had the medical history that I did, with losing a second pregnancy almost halfway through and nearly losing my first one. It just kind of put those things into different perspective instead of wondering, well, why? (#9; aPLs + SLE)
		I had sclerotherapy done on one leg. At that point in time I did not test positive for what they were testing for, and so everybody was bewildered why, I got blood clots from sclerotherapy because it was like extremely rare. Then I had 3 blood clots over a period of 15 years… then I went to [this] research program and they found I actually did test positive. (#21; APS + SLE)
Healthcare experiences	Delay to diagnosis	After my second child was born, I started to have headaches… and I didn’t go to the doctor immediately… in a year or so I started to go to the neurologist, a family doctor. Then I did MRI, and they sent me to different tests. It took 2 years to have a diagnosis. (#4; APS)
		It was always just put under this bigger cloak of high blood pressure… I don’t think until I met [my current doctor], there wasn’t anyone who took all those pieces and put them together. (#5; APS + SLE)
	Feeling unheard	I was having mini strokes for 6 months before I had my big one and everyone just wrote it off, because a woman in pain apparently does not matter…. everyone would pretty much just tell me to push through… [my doctor] just said it was a panic attack. (#6; APS + SLE)
	Accessibility to specialists	We don’t have the healthcare providers available (#11; APS + SLE)
	Lack of information/resources	Just implications of some of the medications, what are the long-term results of using these medications over time? What are the risk factors? (#3; APS + SLE)
		The first time I heard about it was after I’d come out of intensive care… That was the first time I’d ever been introduced to the term. So yes, I had heard that it could be something autoimmune. They had talked about lupus, but the term antiphospholipid antibodies was brand new to me up until January of this year. (#5; APS + SLE)
		I really think there does need to be more information out there for the average lay person. (#9; aPLs + SLE)
		There was some APS information that they had sent me… just a couple of paragraphs of this is what it is, this is how it affects you. (#12; aPLs + SLE)
		It might be helpful to have other people who are going through this and that you could ask questions to. (#14; aPLs + SLE)
		I want to understand what’s going on. Tell me I have a blood clotting disorder, this doesn’t mean anything, I want to understand what’s happening. I want someone to explain it to me. (#20; APS + SLE)
		It should be shredded into a million pieces… it’s an entire booklet that tells you your life is over. Don’t go anywhere, don’t do anything, sit still, you might die. My experience with this disease is nothing of the sort. (#20; APS + SLE)
Lifestyle impacts	Employment/school	I had to start going to all these appointments and getting blood work done and I was like, I don’t have time for this. And, obviously, all the medical professionals are trying to tell me that hey, if you aren’t healthy, school might not even be a possibility. But you know, at that point you’re just like since it wasn’t directly affecting me in a way that I could tell, I was just like, can you guys just leave me alone. (#19; APS + SLE)
	Social	I almost missed out on celebrating holidays for the first time with [my girlfriend], because I was in the hospital… everything was good, but sometimes you miss out on things like that. (#11; APS + SLE)
Emotional impacts	Fear (e.g., future, family planning, the unknown)	It was scary… it all sounded impossible to me at the time. (#2; aPLs + SLE)
		They kind of put the fear of God into you when you find out… the doctors are like you have to do this, you have to do this, you know? (#8; APS)
		My initial diagnosis they were kind of like you have these antibodies, you’re at high risk for blood clots, good luck! So then, of course, I thought I had a blood clot all the time, and took myself to the ER. (#12; aPLs + SLE)
		The idea of blood clots is scary. So I think that was probably pretty challenging for me. (#14; aPLs + SLE)
	Stress and anxiety	It was really stressful because you don’t know anything, really. And the doctors are always so tied up all the time, so they don’t have time to just sit with you and talk to you about it. To this day, I don’t even know what APS really is. I just know that I have to be on blood thinners and I can’t eat leafy greens. (#8; APS)
		Most of my stress and anxiety came from… I was told to watch out for if your chest feels weird, watch out if your legs get swollen…oh, you could get a blood clot in your brain right? At first the information is kind of very overwhelming. (#12; aPLs + SLE)
	Surprise	It was a shock, because I didn’t know what this illness is, and I was 33 years old and I was back in Romania. (#4; APS)
		Very surprised, of course, because I considered myself a very healthy person (#13; APS)
	Uncertainty/confusion	I think it was just really apprehension, like is this something that’s going to be a big deal, and will it impact other things in my life? Again, ongoing long-term medication, what does that mean? (#3; APS + SLE)
		The problem is, I think, when you read some of the literature a lot of it points to lupus, and I’m like, okay? Well, what if I don’t have lupus? What are my underlying causes? So, all we knew is that I had a blood clotting disorder. But I don’t think there’s anything out there today that would tell you what caused it. What caused my issue and what continues to cause my issue? (#7; APS)
		I guess the hardest part was… your life changes in a minute and then, you know, you’re having to inject yourself, and then you still have this family at home that you worry about, and not knowing what the disease really is. That was the hard part for me…It’s just the unknown, the unknown of the disease. (#8; APS)
		It was really confusing, initially, like it’s a big word. (#14; aPLs + SLE)
	Sadness	I was so sad and crying. (#16; aPLs + SLE)
		Very defeating, I guess, is the best word to say? I definitely struggled. I’m still struggling. I was on anti-anxiety and depression medication beforehand, and then with all of the medications and complications and all the new medications that I had to go through, was very much an up and down process of trying to figure out what I could do. (#6; APS + SLE)
	Relief	It was a tremendous relief, because in hindsight I had difficulty with my pregnancy. (#5; APS + SLE)
		It was an a-ha moment… now the pieces have come together, why I got blood clots from sclerotherapy… When I was diagnosed, I was actually kind of happy that I knew what it was. (#21; APS + SLE)

#### Medical complications/physical symptoms

Participants shared diverse aPL/APS diagnosis experiences. Thrombotic or obstetric complications were reported both around the time of diagnosis and before diagnosis, with detailed data provided in accompanying tables (Table 2; Supplemental Table 1). Uncertainty around how they were diagnosed also emerged (*n* = 5); for example, amongst those with aPLs or who received a diagnosis following bloodwork.

#### Healthcare experiences

Identified healthcare challenges around diagnosis included experiencing a delay to diagnosis (*n* = 8), feeling unheard by healthcare providers (*n* = 3; *everyone would just tell me to push through… [My doctor] said it was a panic attack (#6; APS+SLE)*), and limited accessibility to specialists (*n* = 3). Ten participants described their lack of aPLs/APS knowledge at diagnosis, and many (*n* = 10) shared that they were not provided resources to help them through diagnosis. Amongst participants that reported receiving resources, physician support (*n* = 7), handouts (*n* = 5), and websites (*n* = 1) were identified. When asked about suggestions for resources at diagnosis, most (*n* = 13) emphasized the need for additional aPL/APS information. Specifically, medication-related information *(Being able to work with somebody who knows how warfarin works in relation to vitamin K (#8; APS)), information from credible sources (something that’s been approved by trained physicians (#19; APS + SLE)),* examples to help contextualize management behaviours (*Can I sit on the couch and watch a movie?… more situational examples… (#12; aPLs+SLE*)), and information for those without SLE (*A whole different resource instead of always focusing on the lupus patients (#7; APS)*) were identified. Other suggestions included ensuring resources are produced in lay language, avoiding fearmongering (*it’s an entire booklet that tells you your life is over. Don’t go anywhere, don’t do anything, sit still, you might die. (#20; APS+SLE)*), and support groups.

#### Lifestyle impacts

Challenging lifestyle impacts around diagnosis were also identified (It was 1 month going to the doctor almost every day (#16; *aPLs + SLE*)). Specifically, challenges balancing employment/school (*n* = 4) and social impacts (*n* = 4) emerged:

Having all your family members being like, ‘what’s wrong with you? It can’t be that bad.’ (#8; APS)

#### Emotional impacts

The emotional burden of diagnosis emerged (It’s been a traumatic experience (#7; APS)). Seven participants identified fear, particularly related to future impacts, family planning, and the unknowns surrounding aPLs/APS. Similarly, participants shared feelings of stress and anxiety (*n* = 6), associated with their medical complications or lack of knowledge about aPLs/APS. Although surprise (*n* = 5), confusion (*n* = 4), and sadness (*n* = 2) were articulated, feelings of relief were also shared (*n* = 5), particularly as participants identified that the diagnosis helped ‘*explain some things that happened in my past (#9; aPLs + SLE)*:

Discovering there was an underlying cause… was really quite comforting. (#5; APS + SLE)

Two participants (both with SLE) reported that they experienced no emotional impacts as the aPL/APS diagnosis ‘*was never a shock’ (#11; APS + SLE)*.

### Daily life

Participants described everyday experiences of living with aPLs/APS. Primary themes included: physical and psychosocial health, relationships, leisure/lifestyle activities, paid employment, and family planning (Figure 1; Table 3; Supplemental Table 1).

**Table 3.** Experiences of Living with aPLs/APS.

Primary themes	Sub-themes	Illustrative quotes
Physical and psychosocial health	Physical impacts	It was crusty rashes all over my face and hands, which I’m going through again right now. (#1; APS + SLE)
		I went off the blood thinners, I developed bilateral DVTs. So, then I was put back on blood thinners, and they said probably you’re just going to have to have a lifetime membership. (#3; APS + SLE)
		I was given blood thinners after my second surgery for cancer, because I did get a clot, and an abscess after the first surgery. (#9; aPLs + SLE)
		Tiredness has been a huge problem, and again I don’t know if that’s specifically anemia and the other stuff that’s going on, or if antiphospholipid has contributed to that. (#14; aPLs + SLE)
		You put on that bathing suit, and [the leg is] all I see…That’s a big part of it, psychologically, I guess. And the leg does hurt. As the years go on it hurts more. I wear compression socks… I think it’s more cosmetic. (#21; APS + SLE)
	Worry about future	I’ve always been scared of my mortality and death. And being diagnosed with this, it feels like I almost have a timer above my head for my expiration date… makes me feel like if I’m not doing everything to the fullest I’m wasting my life. (#6; APS + SLE)
		I often think that 1 day I’m just going to die of an aneurysm, like that’s unfortunately what I feel like. I do often think that I’m not going to be living to be super old, because I’m already on warfarin, and have been for 10 years. (#10; APS)
		Is it going to kill me 1 day?… I don’t know what’s going to happen? So just the fact that I don’t know if it’s going to affect me. (#15; aPLs + SLE)
		You just have to wait and see, for this catastrophic event to happen, you know? So, there’s always that in the back of your mind when you’re thinking of it. (#17; APS)
	Stress and anxiety	I guess just the uncertainty about my INR levels all the time, and you know, maybe I shouldn’t have that spinach salad. I should have a lighter green salad or something. So just doing things to keep my INR at a good level. The worry that I’m forming another clot… and not having my INR at a good level. (#1; APS + SLE)
		It’s just trying to keep that balance of not getting too stressed out about all those… worst case scenarios. (#3; APS + SLE)
	Feeling like a burden	Everything that occurred so far has been stressful, especially even if not in my eyes, for the people who have had to deal with me. I’m a burden, right? It’s now who’s going to drive me where? Who’s going to do this? Who’s going to do that? (#11; APS + SLE)
	Other psychosocial impacts	I’m pretty optimistic. I like to think I have a positive outlook. I don’t know if everybody who is in my position feels the same way. (#14; aPLs + SLE)
		I feel like I was lucky I did not experience anything since my diagnosis… if I start having any symptoms, I don’t know what the symptoms would be even? (#15; aPLs + SLE)
Relationships	Family, friends, and partners	I was previously married, and that wasn’t a supportive environment. So, my current situation is definitely supportive and my kids are very supportive as well. (#3; APS + SLE)
		My eldest sister has definitely been my rock for anything health-related. That’s the one who is a nurse. So, any problems or anything that I’m really worried about, I always go to her. (#6; APS + SLE)
		My partner always sticks up if something’s not safe… which is a pro and a con cause it feels like, especially with COVID, I was kind of trapped in a bubble, which was both a good thing and a bad thing. I felt safe, but I also felt trapped. (#6; APS + SLE)
		Anytime that I would get really upset thinking about the complications for having babies the most common response is ‘that’s not the only way to have babies’, which is frustrating. (#6; APS + SLE)
		It’s affected my husband and I, like how intimate we are. (#8; APS)
		A lot of people don’t even know what APS is. I didn’t know what it was. And then you kind of feel like the elephant in the room sometimes…and I’ll go to take a drink of, say, a sip of wine or something, and they’re like, well, can you even do that? (#8; APS)
		I think my girlfriend got the biggest blow from all of it… She was shell shocked, it’s a change. It’s a change that I don’t think anybody should really go through. (#11; APS + SLE)
		Honestly, I haven’t told my family or friends about it. It hasn’t had any effect. (#15; aPLs + SLE)
		I think my mom just gets more worried, especially if she’s in an appointment and they ask, are you planning for kids? Because she’s like, my God! What if she can’t have kids, or, you know, all those thoughts kind of come up?… I only have brothers. My older brother is a lot more like protective, if we are play fighting I’ll hit my little brother. It’ll be like a small punch, and if he tries to punch me back my older brother gets very protective just because he’s like, you know, she bruises easily! She’s on blood thinners! (#19; APS + SLE)
	Support provided	[My sister] has taken me to all my appointments. She makes sure that I take my warfarin, and so I feel more dependent on her than I like. (#1; APS + SLE)
		I have a really great partner, I’m really lucky in the sense of my support system in general is outstanding. Everybody is so understanding, and they do what they can to help me out. (#8; APS)
		There’s tons of things that people could do to be more supportive. But people don’t like sick people, and I am chronically sick, and there’s nothing I can do about it. (#20; APS + SLE)
Leisure/lifestyle activities	Impacts on physical activity	I used to be a very active triathlete, and so now cycling is a worry for me to go, not in the basement, but to go out on the road like I used to, and I think that’s more with just being on blood thinners. (#1; APS + SLE)
		I’m looking for another sport. And I’m thinking I’m going to take up swimming. I’m hoping that’s going to be okay. So, I’m hoping to continue hiking. But I’m not going to be able to do the big mountains. (#13; APS)
	Travel	When we went on a road trip I might have taken an extra stop to make sure I was walking around. (#12; aPLs + SLE)
		I used to be a very keen traveler, but not nowadays, you know? I don’t know about the hospital systems in other places, and not that I think I’m going to be there. But it is a concern for me. (#13; APS)
		The idea of a 24h flight is crazy now. (#14; aPLs + SLE)
	Hobbies	I sing in a choir, and I coach a soccer team, and none of those things have changed. (#5; APS + SLE)
		When I get tattoos, because anyone in my life who knows me knows I love tattoos. I just have to be really careful because they scar funny now, and they bleed a lot. (#6; APS + SLE)
Paid employment	Altered career trajectories	I used to have a shop with my sisters. I had my own business and I would easily get overwhelmed and out of breath. I should be able to lift 50lbs, and I can’t anymore. It’s straining. I get dizzy. It takes way more of a toll than it should on my body. So, I am actually currently unemployed because I am too afraid to go back out in the workforce and possibly have something bad happen. (#6; APS + SLE)
		I’ve been living with lupus and LN for quite a long time, so this [the aPL] is not something that’s going to stop me… I’m still going to continue working until the day I can’t (#15; aPLs + SLE)
	Time missed	I’m on disability… I’ve never been on disability in my life. I’ve never not worked in my life. So that’s been a very big change for me. (#1; APS + SLE)
		I haven’t been able to work for a long time, but it’s not because of APS. It’s lupus, but is that APS? Nobody knows. (#20; APS + SLE)
	Accommodations provided	We have the option to go to the office or work from home. Of course, when I need to go, I go. But when I’m not feeling good. I just say, okay, I’ll be working from home today. And that’s fine, they are very flexible. (#16; aPLs + SLE)
Family planning	Decision-making impacts	I had a very high-risk complicated pregnancy… I got a tubal ligation to you know, just resolve that anxiety. It was a bit of a hard decision. But I just felt for my situation, it would be high risk to get pregnant again. (#3; APS + SLE)
		So, after I had my daughter I saw doctors afterwards. That’s when the reality of how bad it could have been came out… I actually went on to have a hysterectomy… I had to make a decision, and that was the best decision for me, because I didn’t want to put myself at risk now that I had a child. (#10; APS)
		I did have a lot of miscarriages… It was difficult… the one that in particular told me to stop was when the fetus made it to 18 weeks and then died. (#13; APS)
	Miscarriage and other complications	It’s an emotional roller coaster, for sure. I did turn 50 last October. So, you know the chances of having kids of our own would be slim to none, probably now… And they would tell us back in the day there are things that we can do to help support and monitor, but you know the third pregnancy, obviously I wasn’t monitored quick enough, and the fetus died sometime inside me, I couldn’t tell and when we went to get our ultrasound there was no heartbeat. (#7; APS)
		The doctor comes back in with nurses and tells you the fetus has died… this is too much mentally (#13; APS)
	Medication experiences	It was just one more thing that I kind of had to deal with, but I knew it was necessary, so I mean I didn’t particularly enjoy it, but it’s like that’s what I had to do for a healthy pregnancy, so I just had to protect myself as well. So, I just looked at it that way. (#3; APS + SLE)
		I knew that it helps to keep the pregnancy, I would do it in a second anytime. (#4; APS)
		There was no choice, because warfarin was not safe for pregnancy, so I had no option but to go on the needles for whatever period of time. The duration before planning in one case, and then during pregnancy, and then even for a period afterwards, you still have to be on these needles, and then they would transition you back once the whole process was done and over with, back to your warfarin. (#7; APS)
		The first factor was the cost. So, the doctor at the fertility clinic was outright like these are super expensive. So, you need to decide if you want to pay for these and keep in mind I had already suffered 3 miscarriages so of course I want to pay for these, it just seemed like such a callous way of presenting it. (#10; APS)
		It wasn’t a fun experience, like this was something I had to do every day, and sometimes I would have these bruises so it also made it awkward when I would like go for ultrasounds… because they automatically assumed I was being domestically assaulted because I had bruises in a hidden spot on the belly… so, it was always very awkward, and I always felt like I had to explain. (#10; APS)
		I didn’t think it was that big a deal, the needles were small. It didn’t hurt that much, and you kind of schedule yourself and do it the same time every day… I mentally prepared myself for it you know, I have to do this because I’m pregnant, and so I didn’t find it that bad. (#18; APS + SLE)
		I just took it because they told me at the hospital. And then again, my OBGYN said it would be a good idea. So, I’m trusting in the doctors, and so they said that would be best. So that’s what I did. (#18; APS + SLE)
		I did not think about not taking it because we’d already lost a baby. (#20; APS + SLE)
	Lack of resources	I don’t know if I was really given that much information about it. It was just this is what it is, and this is how we have to treat it… I think the doctor shared as much information as they had, but it’s not like there was, I don’t recall a big discussion about it. It was just like this is kind of what it is, and this is what we need to do to prevent it. (#3; APS + SLE)
		They were like, okay, you have to stop taking birth control, like yesterday. So no more of those ever and here is a contact with the clinic, and they’ll give you more information. That was pretty much all I got. (#14; aPLs + SLE)

#### Physical and psychosocial health

Physical symptoms that impact daily life were described (*n* = 12). Participants acknowledged that symptoms could result from other co-morbidities (*it’s always APS* vs *SLE (#3; APS+SLE)*); this was emphasized by a participant with APS and SLE who shared that ‘*it’s really hard to tell what’s left over from my stroke. I don’t know if I would count that as the same as my APS? (#6;APS+SLE)*

The most frequently mentioned psychosocial impact was worry about the future (*n* = 17); fear of mortality, experiencing a ‘*catastrophic event’ (#17; APS)*, or that relatives will be impacted by aPLs/APS were articulated. Stress and anxiety (*n* = 13) emerged, particularly around the potential for medical complications, participating in everyday activities (*cycling is a worry… related to being anticoagulated, and having a fall (#1; APS+SLE)*), and uncertainty around international normalized ratio (INR) levels. Two APS participants shared that they felt like a burden. Other psychosocial impacts (*n* = 9) include frustration, sadness, optimism, and feeling fortunate to have experienced minimal impacts.

#### Relationships

Positive and negative impacts on relationships were described. Impacts on family and friends were shared (*n* = 16); while some relationships were strengthened, others deteriorated:

*My relationship with my family has become stronger, my relationship with the family that I’m potentially marrying into has become weaker*. *(#11; APS + SLE)*

Amongst family specifically, participants shared caring and empathetic relationships, though tensions due to feeling that parents/siblings were acting overprotective emerged.

My mom starts to say ‘oh poor girl.’ (#16; aPLs + SLE)

Relationships with partners were also impacted (n = 10); participants shared supportive experiences as well as tensions that led to impacts on intimacy and even separation:

When I was pregnant my partner was not super supportive.(#10; APS)

Finally, participants described how relationships with children were impacted (*n* = 5). Participants shared worries that children would be impacted by aPLs/APS, feeling like they are overprotective (*he doesn’t want me to drive (#13; APS))*, and altered parent-child interactions:

I’ll bump myself and I’m bruised for months and my kids are like ‘Mummy, what is that?’ (#8; APS)

While emotional, transportation, and financial support from family, friends, and partners were described (*n* = 14), examples of feeling unsupported also emerged (*people don’t like sick people, and I am chronically sick (#20; APS+SLE))*. Four participants reported feeling socially excluded due to their illness:

Being social is more difficult... I don’t want to go out as much. (#1; APS + SLE)

Participants (*n* = 5) also shared that due to the rare nature of aPLs/APS, family/friends lacked understanding and are unsure of how to provide support:

They don’t understand… it isn’t out there, and it’s really an unknown thing. (#9; aPLs + SLE)

Three participants described that relationships are not affected, and one (aPLs + SLE) indicated that they had not told family or friends as it has not affected their life.

#### Leisure/lifestyle activities

Participants also described altering physical activity (*n* = 7) due to risks associated with taking anticoagulants, physical symptoms (**e.g.**, dizziness), or potential unknown impacts:

Before my stroke [I did] an aerial silks class. I can’t do that anymore… if I’m hanging upside down something could happen. (#6;APS + SLE)

Similarly, participants reported impacts to travel (*n* = 5) due to sedentary periods in transit, challenges securing travel insurance (e.g., while taking warfarin), or the unknowns related to hospitalization in another country. Other impacts on hobbies (*n* = 2), and sedentary behaviours (*n* = 1) were described *(going to a restaurant and sitting down for an hour (#14; aPLs+SLE)*). Seven participants, with aPLs and APS, reported no impacts to leisure activities.

#### Paid employment

Ten participants (*n* = 4 aPLs, *n* = 6 APS) experienced no impacts to employment due to aPLs/APS as they were already retired when diagnosed, have experienced no symptoms, have not missed time at work due to symptoms, or felt supported by employers. Seven described time missed due to thrombotic events, surgery (e.g., heart surgery), being on disability, or maintenance behaviours (e.g., bloodwork). Altered career trajectories were reported (*n* = 7), though difficulty differentiating between impacts of APS versus other co-morbidities was acknowledged:

I have lupus and APS, it’s hard to separate those... Probably it has affected certain career choices, just more with the fatigue, and you know APS is part of that. (#3; APS + SLE)

Ten participants felt that employers and colleagues were understanding, and six described accommodations (e.g., working from home, taking time off). Despite feeling supported, one participant articulated the need to ‘*overcompensate so it didn’t create an issue’* and the implicit challenges associated with chronic illness:

Companies say they’re supportive but… if they had to choose between two people, who would they choose? Someone who’s never had issues. (#3; APS + SLE)

#### Family planning

Past and future impacts on family planning were discussed. Six participants shared they had never been pregnant; their aPLs/APS diagnosis was identified as one factor in this decision:

One of the reasons we decided to not have any children was lupus at first, but this new diagnosis also. (#15; aPLs + SLE)

All other female participants had been pregnant; four became pregnant following diagnosis, and one was diagnosed during pregnancy. Amongst those who were pregnant prior to diagnosis (*n* = 9), experiences of pregnancy complications impacted family planning:

Because of the issues… we’d always wanted four kids, we stopped after two. (#9; aPLs + SLE)

Experiences of miscarriage (*n* = 5) and other complications (*n* = 5) were discussed (*I had a blood clot go through my lungs (#18; APS + SLE)*), and participants shared how aPLs/APS impacted decision-making. Specifically, fear of pregnancy impacts (*n* = 5) and passing on their illness(es) (*n* = 4) were identified (*I would be doing a disservice to a future child (#14; aPLs + SLE)*). Two participants (both with APS) underwent tubal ligations after experiencing pregnancy complications. Eight participants identified that aPLs/APS did not impact family planning; amongst those who shared why, they identified age at diagnosis, that they were not yet considering having children, or that they did not want children regardless of their diagnosis.

Challenges during pregnancy with LMWH were described. Seven participants identified using injectable blood thinners; while two ‘*didn’t think it was a big deal’ (#18; APS + SLE)*, physical impacts (e.g., pain [*n* = 3], bruising [*n* = 2]) and stress and anxiety (*n* = 3) around taking the shots were described. Six indicated they ‘would do anything’ (#4; APS) for a child and had no choice to maintain their/their fetus’ health. Nine participants had not been provided resources to help them through family planning with aPLs/APS.

### Healthcare and treatment

Interviews concluded with a discussion of aPL/APS healthcare and treatment. Primary themes included: medication experiences, other management behaviours, decision-making considerations, and gaps in care (Figure 1; Table 4; Supplemental Table 1).

**Table 4.** Experiences of healthcare & treatment.

Primary themes	Sub-themes	Illustrative quotes
Medication experiences	Lifestyle impacts	I do blood tests every 2 weeks, so that’s something that impacts. If I’m going away, what does that look like? (#3; APS + SLE)
		I have to go for blood work quite often, because I’m on the warfarin. I’m still going for blood work every second week. So, the inconvenience of getting that blood work done, and we’re currently going through a change of lab services, and the new lab is just a nightmare. I had testing done yesterday in our local lab even with the appointment it took an hour to sit through. (#5; APS + SLE)
		The lab situation for testing INR is not great. I was really having difficulty booking in the exams when they needed me to go in, and because I have my daughter and I’m the primary caregiver, I can’t really go just at any given hour… My INR varies a great deal still, so often I’m testing once a week and so it’s continuously trying to monitor foods, amount of exercise, and then sometimes feeling really defeated because I have no idea why it’s gone down, or it’s gone up. (#10; APS)
		Learning how to coordinate with all of my other medications and making sure I’m not overlapping was an adjustment I had to make. (#14; aPLs + SLE)
		I’ve gotten to a point of being used to needles and stuff. So, it’s fine. And you know, you get a system figured out. (#21; APS + SLE)
	Side effects	It was really crappy on my body, because sometimes I’d feel really sluggish and slow, and other times I’d be covered in bruises, and I felt like there was no in between. (#6; APS + SLE)
		One of the other things that’s really affected me is with my period, it got so bad that I couldn’t even leave my house for almost 10 days. So, I ended up getting an IUD, which I swore I would never get one of those, but I had no other options… before that I would have a period for like 3 days, and it would never slow me down. But since being on blood thinners, I had to do something because it was totally taking over my life. (#8; APS)
	Physical burden	They’re really painful. I take a lot of needles, but I would say that the blood thinners hurt more than most of the other stuff, and they hurt for a while. (#14; aPLs + SLE)
	Emotional burden	I’ve had a clot so you know we’re trying to prevent it from getting bigger, so the importance of that overrides me being a chicken. (#2; aPLs + SLE)
		I can’t inject myself, I’m terrified of needles. It’s the only thing I’m terrified of so of course I had to be on them. But no, my husband, he would do them for me… I think I only injected myself twice because I had to, but I was bawling my face off the two times I had to. (#8; APS)
Other management behaviours	Healthcare providers	I see a rheumatologist, a hematologist and then just my general practitioner. (#19; APS + SLE)
	Diet	I have to monitor my food intake, and make sure that I get the right amount of vegetables. My husband always says we have too much vegetables and too much exercise. We have to make sure we have vegetables and exercise, and then my levels have been really good, so mostly it’s the management with the warfarin. (#5; APS + SLE)
		Because I’m on the warfarin, I just have diet restrictions… So, I just have to be really cognizant on my diet plan. I do see some specialists that try to put together some diet plans for me that work. Mind you, it’s not always the food I like. (#7; APS)
	Physical activity	I was at a local YMCA to proceed with an exercise program, because I know that would help, especially… to deal with all these potential consequences. (#11; APS + SLE)
		Exercise, that’s just for my whole wellbeing. But it’s the blood circulation and I think that’s a very healing thing. Yeah, for the leg as well. (#21; APS + SLE)
	Rest	In theory, if I was taking proper care of myself, I would try to get a good night’s sleep, have a healthy balanced diet, drink lots of water, make sure I’m getting physical activity in, I am just not good at it. (#12; aPLs + SLE)
	Other behaviours	I participated in recreational marijuana before, so they told me don’t smoke it because it causes stress in the lungs. (#12; aPLs + SLE)
		I try to keep my mind positive and say, I’m okay. That’s my illness, it’s not the end. I try to follow the instructions of my doctor and I try to spend time with my family and I try to be happy almost every day. (#16; aPLs + SLE)
Decision-making considerations	Role of healthcare providers	I just listen to her 100%, just whatever she thinks I should take… She just right away put the pieces together and put me on the Plaquenil and the Cellcept that I’m on now, like she nailed it right off the bat, like the dose hasn’t changed. Yeah, I trust her implicitly, and I think she’s just amazing. (#2; aPLs + SLE)
		I’ve always been fortunate to have doctors that don’t want me on any more medications than I absolutely have to be on. (#21; APS + SLE)
	Lack of options	I didn’t even get to make the decision, they just put me on it, and they were like this is what you have to take from now on. (#6; APS + SLE)
		I don’t think there was any consideration. It was pretty much take it or die. (#11; APS + SLE)
	Medication side effects	I was very worried, because, if you don’t know anything about warfarin and you read up there’s lots of things that are like, it’s like rat poison, and you’re going to get stomach ulcers. And so, I was really scared. (#10; APS)
	Coverage/financial	Finding out how much they cost. That was another thing. It’s a lot of money. Because we didn’t have health coverage and all of a sudden, it’s like you have to spend $1100 a month for this. (#8; APS)
Gaps in care	Systemic challenges (e.g., uncoordinated care, wait times, accessing system)	Health care in general needs to be adjusted. Waiting three and a half hours, because you potentially might be having a heart attack in the Emergency Room is a danger… There’s a reason why I have to tell triage every time I go in, everything wrong with me, because if I don’t, I don’t get in in time. And there will be other issues that occur… just in the general sense, the ability to have more doctors in an area… no one is accepting patients. (#11; APS + SLE)
		Uniformity of care would be nice. Like the way that the anticoagulation clinic treats blood thinners and calling patients is different than my general practitioner’s way. Which also might have been a reason why my INR got a little out of control for a second, but just kind of like consistency across practices. (#19; APS + SLE)
	Communication	Maybe just open lines of communication about like, hey, can I ask you some questions about this thing you sent me?… I know people are busy. I hate to be like I’ll put more work on this person. But if you are not a scientist and you read a scientific study, 99% of that is going right over your head. Like you’re not going to understand the terminology, you’re not going to understand niche terms and stuff like that…that has been a big struggle for me throughout my illness journey. (#14; aPLs + SLE)
		My doctors right now are doing a good job. I would say that in the past my doctors could have looked me in the eye, at least pretended to care, or at least made it appear to me like they cared. (#20; APS + SLE)
	Lack of aPLs/APS information/resources	Having a support hub where there’s information, or people with antiphospholipid could go on or ask questions, or get help. Even from information booklets that we could actually trust and not just take a gamble being like, “I think this information is correct”. I think it would help a lot, because yeah, we don’t always want to bug our doctors every 2 seconds if something weird comes up. But it’s still nice to be able to get consistent accurate information. (#6; APS + SLE)
		I went to a pharmacist by the hospital I went to, which is in another small little town and he’s like, why are you on warfarin? So, I told him I have APS. He goes, well, what is that? And I said, well, like my blood clots. It’s just basically what I say. And he goes. Okay, well why don’t you get the CoaguChek? And I said, well, I heard it’s not accurate for APS, and he goes well no, it’s accurate for everybody. But then you talk to my [other] pharmacist and she goes, they’re not accurate for APS. So if you had definite research on that, that would solve the issue with having to go get your blood checked all time. (#8; APS)
		If I wasn’t working with [my current rheumatologist], I would for sure say that they need to be way more informative to their patients about APS, because even the hematologist in [my old city] to this day, I haven’t got any information from him… I would say they need to make their patients more aware and just spend the time and answer the questions. Otherwise, you create so much panic. (#8; APS)
		Lupus Canada has a library of articles that you can tap into, and you know what you’re getting is legitimate. It’s not some junk off the Internet, it’s all legitimate articles. And so, in that way a library of legitimate articles that you can tap into and download would be awesome. (#9; aPLs + SLE)
		I feel like a lot of people don’t know what this is, I’ve had to actually when I’ve gone into to Emerge and I’m being triaged, I’ve actually had to spell it for them, they have no idea what this is sometimes… then you’re also trying to explain the reason this week-long migraine where I can’t open my eyes is so serious is because I have this clotting condition, and trying to impart that information and get them to understand it, because otherwise what they’re writing down is she has a headache… What ends up happening is you’re triaged at the bottom of the list, because they don’t know what it is, they don’t have the time to look it up, even though you’re giving them the information. (#10; APS)
		It would be great if there was like this place that had all the information, and they were like, or here, there’s this book. I would go buy it, or I would go get it out of the library, whatever. But I don’t even think I got a brochure to be honest. (#10; APS)

#### Medication experiences

Various aspects of healthcare and treatment were discussed, particularly around the burden associated with medication use. All participants (*n* = 21) shared experiences of managing their aPLs/APS with medication, including aspirin (*n* = 12), warfarin (*n* = 11), LMWH (*n* = 6), and other medications (*n* = 11).

Medication-related lifestyle impacts (*n* = 14) were described. The challenges of coordinating medication (*My life is almost ruled by my medication (#6; APS+SLE*)), monitoring diet, and the frequency of appointments for measuring INR were emphasized; challenges with lab accessibility (e.g., wait times, communication) were highlighted. To reduce the lifestyle burden associated with lab visits, four participants identified the potential for at-home INR testing. While one had purchased an at-home testing device, hesitation around accuracy emerged (*n* = 2; *I’m not sure I’d trust that (#1; APS+SLE)*).

Medication side effects (*n* = 9) were also discussed (*Warfarin itself is a good medication. I’m annoyed that I can’t get tattoos very often (#11; APS+SLE)*). Similarly, the physical burden (e.g., pain/bruising related to taking injectable blood thinners) (*n* = 6) and measuring INR (*n* = 2) was shared. The medication-related emotional burden (*n* = 7) was described; participants shared the ‘constant reminder of disease’ (#20; APS + SLE), fear of needles, and stigma associated with warfarin use.

#### Other management behaviours

Participants reported seeing rheumatologists (*n* = 19), hematologists (*n* = 14), family physicians (*n* = 11), and other healthcare professionals (*n* = 10; nephrologists, pulmonologists, gynecologists, dermatologists, physiotherapists, neurologists) for their aPLs/APS. They identified physical activity (*n* = 7) for both overall health and to ensure blood circulation, monitoring diet (*n* = 4) for overall wellbeing, and rest (*n* = 2). Six participants shared other behaviours including monitoring sleep posture, meditation, smoking cessation, avoiding stress, maintaining a positive mindset, and avoiding high-impact activities.

#### Decision-making considerations

When asked about factors that contribute to medication decision-making (e.g., changing/starting a new medication), the role of healthcare providers was most frequently identified (*n* = 17). Some participants felt they had no choice (*n* = 5; *it was pretty much take it or die (#11; APS)*), and others (*n* = 4) described the role of potential side effects (*I don’t want to create more issues than I already have (#7; APS)*). A lack of coverage (*n* = 2), and other factors (*n* = 7) including family planning considerations, safety, time, choosing the mildest possible medication, and balancing medication with other lifestyle factors were also identified:

As I start going to work… if I forget it more often, how much does that increase my risk? (#14; aPLs + SLE)

#### Gaps in care

Most participants (*n* = 18) expressed an overall positive impression of their care, though concerns regarding certain healthcare providers were articulated (*[My family doctor]’s not as invested as he used to be (#6; APS + SLE)*). When gaps in care were discussed, systemic challenges (*n* = 6; e.g., uncoordinated care) were shared. Though many (*n* = 10) spoke positively about communication with physicians, some (*n* = 6) highlighted communication-related challenges:

Doctors treat us like infants, like we can’t be intelligent enough to be the head of our own medical team. (#20; APS + SLE)

When asked about other suggestions to improve care, ten participants indicated they did not have enough information about aPLs/APS; the need for increased accessibility to information (*n* = 8) for themselves (*You’re just sort of paddling blind (#13; APS)*), society, and healthcare providers (*I would like to have a [family] doctor that knows this syndrome (#4; APS)*) was shared. Finally, the need for additional emotional support/support groups (*n* = 7), and accessibility to healthcare providers (*n* = 7) were identified *([My physician’s] just like… ask your specialists (#6; APS + SLE))*.

## Discussion

This study explored patient experiences of aPL/APS diagnosis, daily life, and healthcare and treatment; participants highlighted how the diverse manifestations of aPLs/APS, management-related challenges, family planning experiences, and lack of aPLs/APS supports/resources impose considerable physical, lifestyle, and psychosocial impacts on themselves and their social networks.

While this research is unique in its qualitative exploration of the lifecosts^32^ of aPLs/APS, the physical, psychological and emotional impacts identified are similar to those in qualitative studies assessing patient experiences of venous thromboembolism.^36^ For example, the shock experienced at diagnosis,^36–38^ fear of future,^36,39^ impacts to daily life,^36,38,40^ and the need for additional information^36,41^ have been documented amongst those with thrombotic complications more broadly. Participants also shared the burden associated with anticoagulation; challenges around managing diet, maintaining INR levels, and frequent laboratory visits have been described elsewhere.^42^ While the emotional burden and lifestyle impacts (e.g., on family planning) echo results from previous qualitative studies with SLE^32^ and LN^35^ patients, experiences ranging from minimal physical or lifestyle impacts to major thrombotic/obstetric complications emerged in our results due to the diverse manifestations of aPLs and APS. Healthcare providers should be aware of the emotional burden associated with an aPLs/APS diagnosis regardless of a patient’s disease severity; most (*n* = 17) participants articulated worry about their future following diagnosis, even if they had not experienced a clinical event.

Our sample included patients with aPLs and APS, with/without SLE. While our analysis did not account for differences between groups, the varied experiences of those with aPLs and APS should be acknowledged. For example, diagnosis experiences varied amongst those with aPLs and APS. Some participants had received an APS diagnosis following a thrombotic/obstetric event, while some with aPLs described uncertainty around how they were diagnosed, or were diagnosed based on bloodwork alone. Despite the varied diagnosis experiences, the emotional burden of an aPL diagnosis (e.g., anticipatory fear) remains; *of course, I thought I had a blood clot all the time (#12, aPLs + SLE*). Similarly, participants with aPLs and APS shared worry about future impacts; for some with APS, this was rooted in previous catastrophic events, and having to ‘*wait and see*’ if complications arise (*#17; APS)*. Those with aPLs may never experience medical complications,^43^ making the ‘wait and see’ experience particularly unique. Some participants reported few or no lifestyle impacts; for example, 66.7% of those with aPLs reported no impacts to paid employment due to aPLs. This is unsurprising as individuals with APS or aPLs/APS and SLE are more likely to experience a higher burden of illness resulting in more substantial lifestyle impacts.

Impacts on pregnancy and family planning were a critical component of the aPL/APS lived experience. Experiences of pregnancy in SLE,^44^ and in patients with aPLs/APS^21,28,29^ have previously been explored; feelings of uncertainty (e.g., around future pregnancy) and the lack of APS information that emerged in previous studies^28,29^ were consistent with our participants’ experiences. Further, attitudes around anticoagulant use were similar to previously documented APS patient experiences of LMWH in pregnancy^21^; participants expressed they would do anything to ensure a healthy pregnancy, and described *‘trusting in the doctors’ (#18; APS + SLE)* when making medication-related decisions.

Participants’ experiences with aPL/APS supports and resources were limited. A lack of information/resources was reported at diagnosis (*n* = 13), during pregnancy/family planning (*n* = 9), and around medication decision-making (*n* = 10). This is consistent with studies on patient experiences of other autoimmune disease, where participants have reported a lack of information amongst patients,^35,45^ in society,^35,45,46^ and compared with other chronic illnesses (e.g., diabetes^35,46^). Given the lack of resources and the reported medication experiences (e.g., lifestyle impacts, emotional burden), patient decision-making aids around treatment options are needed. Amongst the available resources, participants requested more lay language information, medication-related information, examples to help contextualize management behaviours (e.g., situational examples about sedentary behaviours, information regarding at-home INR testing devices), and targeted information and resources for both those with aPLs and APS with and without SLE. These findings suggest a critical need for advocacy organizations to develop and disseminate more patient resources. Increasing the availability and accessibility of credible aPLs/APS information and incorporating the patient perspective when designing and disseminating resources should be prioritized to ensure supports/resources are meaningful to those affected.^47^

While our interview guide asked about patient experiences of aPLs/APS, participants shared experiences related to SLE and other co-morbidities. This may stem from the complexity of the disease and how its manifestations often overlap with other illnesses. The multimorbidity experience has been echoed in other qualitative work,^35,48^ as patients do not necessarily make sense of their illness(es) based on clinical diagnoses alone.^49^ Interactions between chronic illness(es) and their treatments can compound the physical and psychosocial burden, emphasizing the value of person-centered versus single disease-oriented management approaches.^50^

Results of this study are not generalizable given the qualitative approach and sample size, however the objective was to provide an in-depth exploration of the broad experiences of individuals with aPLs/APS. This study also has several limitations. Our results may not fully reflect aPL/APS patient experiences as recruitment occurred from a single APS specialty clinic. Second, while we aimed to sample for maximum variation and include participants with diverse demographic, obstetric and thrombotic experiences, our sample included one male and only 19.1% were of non-white race/ethnicity. Eliciting perspectives from a broader range of patients with varying demographic and clinical characteristics will be important future research. Third, experiences that participants attributed to aPLs/APS may have been due to medication side effects, other autoimmune conditions, or other co-morbidities and not aPLs/APS directly. Fourth, some participants were diagnosed 25+ years ago, and recall bias around early illness experiences may exist. Fifth, while virtual interviews can help ensure participant comfort, reduce geographical barriers and time constraints, and provide other qualitative benefits including documenting facial expressions and body language, eight participants chose to keep their camera off, so body language was not visible during their interviews. Finally, though this exploratory study provides insight into aPL/APS patient experiences, generalizable comparisons between subgroups (e.g., aPLs vs APS; aPLs/APS with vs without SLE) are not possible due to sample size. Further investigating the differences between how patients with various disease manifestations and demographics experience aPLs/APS (e.g., through a large-scale survey) is important.

Through semi-structured in-depth interviews this research paints a more complete picture of the aPL/APS lived experience. Results underscore the use and usefulness of alternative epistemologies for contextualizing the experiences of individuals with aPLs/APS, contributing to the potential for policy and practice change.

## Supplemental Material


Supplemental Material - A qualitative investigation of the experiences of patients living with antiphospholipid antibodies
Supplemental Material for A qualitative investigation of the experiences of patients living with antiphospholipid antibodies by Francesca S Cardwell, Alexandra O Kobza, Susan J Elliott, Paul S Gibson, Nancy Soliman, Leslie Skeith, Ann E Clarke and Megan RW Barber in Lupus.

## Data Availability

To ensure participant anonymity no data are publicly available.*
