# Supplementary material for: A qualitative investigation of the experiences of patients living with antiphospholipid antibodies
Source: Lupus. 2024 Jul 24;33(10):1043–58. doi: 10.1177/09612033241265545 (PMC11977815; doi:10.1177/09612033241265545)
Supplement: Supplemental Material - A qualitative investigation of the experiences of patients living with antiphospholipid antibodies [file sj-pdf-1-lup-10.1177_09612033241265545.pdf]

Supplemental Table 1: Overview of themes and sub-themes and the number of participants identifying each theme throughout qualitative analysis

Themes and sub-themes	Number of participants identifying theme (% of total)
Diagnosis	21 (100.0)
Medical complications/physical symptoms	21 (100.0)
At diagnosis – thrombotic complications	10 (47.6)
At diagnosis – obstetric complications	2 (9.5)
At diagnosis – other physical symptoms	5 (23.8)
In hindsight – obstetric complications	11 (52.4)
In hindsight – thrombotic complications	3 (14.3)
Uncertainty around diagnosis	5 (23.8)
Healthcare experiences	21 (100.0)
Delay to diagnosis	8 (38.1)
Feeling unheard	3 (14.3)
Accessibility to specialists	3 (14.3)
Lack of knowledge at diagnosis	10 (47.6)
Not provided resources at information/resources	10 (47.6)
Supports provided – physician support	7 (33.3)
Supports provided – handouts	5 (23.8)
Supports provided – websites	1 (4.8)

Suggestions for resources – need for additional aPL/APS information	13 (61.9)
Lifestyle impacts	6 (28.6)
Employment/school	4 (19.1)
Social	4 (19.1)
Emotional impacts	21 (100.0)
Fear	7 (33.3)
Stress and anxiety	6 (28.6)
Surprise	5 (23.8)
Uncertainty/confusion	4 (19.1)
Sadness	2 (9.5)
Relief	5 (23.8)
None	2 (9.5)
Daily Life	21 (100.0)
Physical and psychosocial health	21 (100.0)
Physical impacts	12 (57.1)
Worry about future	17 (81.0)
Stress and anxiety	13 (61.9)
Feeling like a burden	2 (9.5)
Other psychosocial impacts	9 (42.9)
Relationships	21 (100.0)
Impacts on relationships with family/friends	16 (76.2)
Impacts on relationship with partner	10 (47.6)

Impacts on relationship with children	5 (23.8)
Support provided	14 (66.7)
Social exclusion	4 (19.1)
Family/friends unsure how to provide support	5 (23.8)
No impacts on relationships	3 (14.3)
Had not told family/friends	1 (4.8)
Leisure/lifestyle activities	16 (76.2)
Impacts to physical activity	7 (33.3)
Impacts to travel	5 (23.8)
Impacts to hobbies	2 (9.5)
No impacts to leisure/lifestyle activities	7 (33.3)
Paid employment	21 (100.0)
Altered career trajectories	7 (33.3)
Time missed	7 (33.3)
Employers/colleagues understanding	10 (47.6)
Accommodations provided	6 (28.6)
No impacts to employment	10 (47.6)
Family planning	21 (100.0)
Never been pregnant	6 (30.0)[a]
Pregnancy experiences	14 (70.0)[a]
Prior to diagnosis	9 (45.0)[a]
Following diagnosis	4 (20.0)[a]
Diagnosed during pregnancy	1 (5.0)[a]

Decision-making – fear of pregnancy impacts	5 (25.0)[a]
Decision-making - fear of passing on illness	4 (19.1)
Decision-making - tubal ligation	2 (10.0)[a]
Miscarriage	5 (25.0)[a]
Other complications	5 (25.0)[a]
No impacts on family planning	8 (38.1)
Medication experiences – injectable blood thinners	7 (35.0)[a]
Not a big deal	2 (28.6)[b]
Physical impacts – pain	3 (42.9)[b]
Physical impacts – bruising	2 (28.6)[b]
Stress and anxiety	3 (42.9)[b]
Would do anything/no choice	6 (85.7)[b]
Lack of resources	9 (45.0)[a]
Healthcare and treatment	21 (100.0)
Medication experiences	21 (100.0)
Lifestyle impacts	14 (66.7)
Side effects	9 (42.9)
Physical burden – measuring INR	2 (18.2)[c]
Physical burden – other	6 (28.6)
Emotional burden	7 (33.3)
Other management behaviours	21 (100.0)
Healthcare providers	21 (100.0)
Rheumatologists	19 (90.5)

Hematologists	14 (66.7)
Family physicians	11 (52.4)
Other	10 (47.6)
Physical activity	7 (33.3)
Monitoring diet	4 (19.1)
Rest	2 (9.5)
Other behaviours	6 (28.6)
Decision-making considerations	21 (100.0)
Role of healthcare providers	17 (81.0)
Lack of options	5 (23.8)
Medication side effects	4 (19.1)
Coverage/financial	2 (9.5)
Other factors	7 (33.3)
Gaps in care	21 (100.0)
Positive impression of care	18 (85.7)
Systemic challenges	6 (28.6)
Communication with healthcare providers - positive	10 (47.6)
Communication with healthcare providers – challenges	6 (28.6)
Lack of aPLs/APS information/resources	10 (47.6)
Need for increased accessibility to information	8 (38.1)
Need for additional emotional support/support groups	7 (33.3)
Need for additional accessibility to healthcare providers	7 (33.3)

[a] *Only female participants (n=20) were included in the denominator for this sub-theme*

[b] *Only participants that had taken injectable blood thinners during pregnancy (n=7) were included in the denominator for this sub-theme*

[c] *Only participants who had reported taking warfarin (n=11) were included in the denominator for this sub-theme*
